# There Is No Community Here: Living Alone, Place, and Older Peoples' Risk of Social Isolation

**DOI:** 10.1093/geroni/igab046.468

**Published:** 2021-12-17

**Authors:** Rachel Weldrick

**Affiliations:** Simon Fraser University, Vancouver, British Columbia, Canada

## Abstract

Existing research has identified significant risk factors for experiencing social isolation in later life including chronic health conditions, mobility impairments, and living alone among others. Although many older people who live alone maintain active social lives, living alone remains a top predictor of social isolation. Less is known about other types of risk factors, such as place-based risks and social exclusion. Despite calls to examine the role of place and social exclusion in social isolation risk, few studies have investigated the links. Models of isolation risk have often omitted place-based factors and social exclusion and focused largely on individual-level risks. In order to address these gaps, this paper presents the findings of 17 in-depth, qualitative interviews with community-dwelling older people who live alone (aged 65-93). Participants were recruited using a theoretical sampling strategy to ensure that a diverse range of neighbourhood types were represented among the participants (e.g., walkable vs. car-dependent neighbourhoods). Interview transcripts were analyzed using a constructivist grounded approach resulting in several major themes. Participants described aspects of their local environments as shaping their risk of isolation including infrastructure and amenities delivered in place, and neighbourhood makeup, among others. These themes are further examined through the lens of place-based exclusion and used to conceptualize how dimensions of both place and social exclusion fit into the model of known isolation risk factors. An adapted model of risk is presented to guide future research and intervention planning.

